# A psychosocial perspective of medication side effects, experiences, coping approaches and implications for adherence in hypertension management

**DOI:** 10.1186/s40885-015-0028-3

**Published:** 2015-09-17

**Authors:** Irene A. Kretchy, Frances T. Owusu-Daaku, Samuel A. Danquah, Emmanuel Asampong

**Affiliations:** Department of Pharmacy Practice and Clinical Pharmacy, University of Ghana School of Pharmacy, College of Health Sciences, Legon, Ghana; Department of Clinical and Social Pharmacy, Faculty of Pharmacy and Pharmaceutical Sciences, Kwame Nkrumah University of Science and Technology, Kumasi, Ghana; Department of Psychology, University of Ghana, Legon, Ghana; Department of Social and Behavioural Health, School of Public Health, University of Ghana, Legon, Ghana

**Keywords:** Hypertension, Medication side effects, Adherence, Ghana, Anxiety, Depression, Stress, Complementary and alternative medicine

## Abstract

**Introduction:**

This study examined whether psychosocial variables influenced patients’ perception and experience of side effects of their medicines, how they coped with these experiences and the impact on medication adherence behaviour.

**Methods:**

A hospital-based mixed methods study using quantitative and qualitative approaches was conducted with hypertensive patients. Participants were asked about side effects, medication adherence, common psychological symptoms and coping mechanisms with the aid of standard questionnaires and an interview guide.

**Results:**

The experiences of side effects—such as palpitations, frequent urination, recurrent bouts of hunger, erectile dysfunction, dizziness, cough, physical exhaustion—were categorized as no/low (39.75 %), moderate (53.0 %) and high (7.25 %). Significant relationships between depression (*x*^2^ = 24.21, *p* < 0.0001), anxiety (*x*^2^ = 42.33, *p* < 0.0001), stress (*x*^2^ = 39.73, *p* < 0.0001) and side effects were observed. A logistic regression model using the adjusted results for this association is reported—depression [OR = 1.9 (1.03–3.57), *p* = 0.04], anxiety [OR = 1.5 (1.22–1.77), *p* ≤ 0.001] and stress [OR = 1.3 (1.02–1.71), *p* = 0.04]. Side effects significantly increased the probability of individuals to be non-adherent [OR = 4.84 (95 % CI 1.07–1.85), *p* = 0.04] with social factors, media influences and attitudes of primary care givers further explaining this relationship. Personal adoption of medication modifying strategies, espousing the use of complementary and alternative treatments and interventions made by clinicians were the main forms of coping with side effects.

**Discussion:**

Results from this study show that, in addition to a biomedical approach, the experience of side effects has biological, social and psychological interrelations. The results offer more support for the need for a multi-disciplinary approach to healthcare where all forms of expertise are incorporated into health provision and patient care.

## Introduction

Hypertension refers to a chronic medical condition relating to an elevation in blood pressure. A review of population-based studies on hypertension in Ghana has showed that the prevalence of hypertension is around 54.6 % in urban and 19.3 % in rural communities [1]. Although worldwide prevalence in the year 2000 was estimated at 26 % totalling approximately 1 billion people, it has been projected to increase to 29 % by the year 2025 due to the expectation that a greater proportion of the world’s population will be older by that year [2]. Hypertension is the leading risk factor for cardiovascular diseases and is a major cause of death globally [3, 4]. Similarly, hypertension is a major risk factor when taking into account death and disability worldwide, and it accounted for 9.4 million deaths and 7 % of disability adjusted life years (DALYs) in 2010 [3].

Although hypertension is an incurable condition, it can be managed with the help of medications and lifestyle modifications. The main goal of treatment is to reduce blood pressure and consequently lessen the risk of complications. Treatment of hypertension is usually based on country-specific guidelines such as the Joint National Committee (JNC8) and the Ghana Standard Treatment (STG6) Guidelines [5].

Medicines such as alpha blockers, angiotensin-converting enzyme inhibitors, angiotensin receptor blockers, beta blockers, calcium channel blockers, central alpha agonists, diuretics, renin inhibitors and vasodilators as well as exercise, weight loss and healthier diet therapies are prescribed in order to achieve optimum blood pressure control [6].

Common side effects associated with the medications include dizziness, fatigue, cough, headache, confusion, depressed mood, chest pain, difficulty breathing, constipation, diarrhoea, swelling in parts of the body, reduced sex drive, erectile dysfunction, persistent cough, increased frequency of urination, rash and difficulty sleeping [6]. Although these adverse effects, as well as common psychological problems like depression and anxiety, have been noted to decrease medication adherence [7–9], some patients have associated and interpreted the effectiveness of their medications with the extent to which adverse events are experienced by them, thereby improving their adherence to therapy [10].

This study therefore sought to examine the role of psychosocial variables in influencing how patients perceived the side effects associated with their medicines, how they coped with these experiences and the impact on adherence to conventional hypertension therapies.

## Methods

### Study design and setting

A hospital-based mixed methods study, using quantitative and qualitative approaches was conducted with hypertensive patients from May to November 2012. The rationale for including the qualitative phase was to triangulate the quantitative phase of the study. The study was conducted at the two major tertiary hospitals in Ghana—Korle Bu Teaching Hospital (KBTH), Accra and Komfo Anokye Teaching Hospital (KATH), Kumasi. The KBTH is the premier and largest teaching hospital located in the Accra District of Greater Accra Region and the second largest hospital in the West African subregion. Being the only tertiary hospital in southern Ghana, KBTH is the main centre for the provision of healthcare for the people of Accra and other surrounding urban towns located southwards. Komfo Anokye Teaching Hospital is the second largest hospital in the country and serves as the main facility for referrals from the northern parts of Ghana. In selecting these two hospitals, it is assumed that research participants with diverse experiences would be recruited to fairly represent both southern and northern parts of Ghana to allow for some degree of generalization of results.

### Participants

Four hundred (400) hypertensive outpatients were recruited from the two hospitals in Ghana using simple random sampling techniques. The minimum sample size was determined using the estimated prevalence of hypertension at the time of study (28.7 %) and a 95 % confidence interval [11]. Patients who were at least 18 years old with a diagnosis of hypertension only or hypertensive with other comorbid conditions were included. Only patients reporting a prescription of at least one antihypertensive medication for a minimum of 2 months were included. Additionally, inpatients, pregnant women (because of the prospect of gestational hypertension resolving after delivery), newly diagnosed patients as well as the physically and mentally incapacitated were excluded from the study.

### Measures

After obtaining informed consent from the 200 recruited study participants from each study site, a researcher-administered structured questionnaire was used to gather data. Information was obtained on demographic characteristics, experiences of medication side effects, negative emotional experiences and medication adherence behaviour.

The Hypertensive Medication Side Effect Experience Scale (HMSEES) assessed the frequency of experiences with various possible side effects associated with different antihypertensive medications. Some examples of the 18-item list of side effects included fatigue, cough, headache, chest pain, difficulty breathing, increased frequency of urination, reduced sex drive and erectile dysfunction. Participants indicated the frequency of experiences on a 5-point scale that ranged from never (0), rarely (1), sometimes (2), very often (3), to always (4) giving a score ranging from 0–72. These were then categorized as no side effects (0), low side effects (1–18), moderate side effects (19–36) and high side effects (37 and above). The HMSEES showed a good reliability of 0.801.

The Depression Anxiety Stress Scale (DASS) is a 21-item self-report inventory that measures the negative emotional states of depression, anxiety and stress. Depression was assessed around themes such as dysphoria, hopelessness, devaluation of life, self-depreciation, and lack of interest/involvement, anhedonia and inertia. The subscale on anxiety measured autonomic arousal, skeletal muscle effects, situational anxiety and subjective experience of anxious affect. In relation to stress, items such as relaxation difficulty, nervous arousal, agitation, irritability and impatience were measured. Participants were requested to use a 4-point severity scale to rate the extent to which they experienced each negative state over the past week. The reliability coefficients for the three scales have been reported as 0.71, 0.79 and 0.81 for depression, anxiety and stress, respectively [12].

Medication adherence was assessed with the 8-item Morisky Medication Adherence Scale (MMAS). The MMAS scores range from 0 to 8 and to be grouped as low adherence, medium adherence and high adherence on the basis of the number of positive responses obtained. Patients who scored low and moderate were grouped as poor adherent. The reliability measure was 0.83 for a study on hypertensive outpatients [13] and 0.793 for this study.

Additionally, an exploratory interaction using one-on-one semi-structured in-depth interviews assessed the views of fifteen participants on side effects, coping and adherence behaviour. The data were saturated when 15 patients had participated in the qualitative study. The interviews were guided by a range of questions on general medication use issues such as (“Is there anything about your medications you don’t like?”) its relation with medication adherence (“Why does this pose a problem to the management of your hypertensive condition?”) and recommendations for improved adherence behaviour (“In your view, what are the possible best ways to solve these problems you stated earlier?”). The moderation was done by one of the researchers. Each interview lasted about 40 min.

### Ethical considerations

The study was approved by the Institutional Review Board at Noguchi Memorial Institute for Medical Research, Accra and Committee of Human Research, Publications and Ethics, Kumasi with the approval codes as NMIMR-IRB CPN 044/10-11 and CHRPE/AP/022/12, respectively. Permission was obtained from the hospital authorities before data were collected. Participation in the study was strictly voluntary and participants enrolled after informed written consent has been obtained from them. Each participant in the qualitative study also consented to the recording of interviews. To ensure that patients and their information were respectively anonymous and confidential, each participant was assigned an identification code which has been used in presenting the results.

### Data analysis

Data from the quantitative phase was analysed with the Statistical Package for Social Sciences (SPSS) version 20 and presented statistically as descriptive and inferential. The relationship between psychological problems, side effects and medication adherence were evaluated using chi-square tests and logistic regression models. The interviews from the qualitative study were audio-taped with a digital audio recorder, manually transcribed and analysed using thematic content analysis. Analysis was performed through a coding process to identify and report patterns. Six phases were involved and this included familiarization with data, generating initial codes, searching for themes among codes, reviewing themes, defining and naming themes and producing the final report. The themes originated from participant interviews a posteriori.

## Results

### Sample characteristics

The characteristics of the patients are summarized and presented in Table 1. Approximately 63 % of the participants were women, 33 % were 50 to 59 years old, 64 % were married, 54 % had obtained secondary school education and 80 % had hypertension for ≥10 years.

**Table 1:** Characteristics of study sample

Variable	Frequency	Percentage
Sex		
Male	149	37.25
Female	251	62.75
Age		
<20	1	0.25
20–29	12	3.00
30–39	20	5.00
40–49	71	17.75
50–59	130	32.50
60–69	105	26.25
≥70	61	15.25
Marital status		
Single	39	9.75
Married	254	63.50
Widowed	73	18.25
Divorced/separated	25	6.25
Cohabiting	9	2.25
Education		
No formal	48	12.00
Primary	33	8.25
Secondary	217	54.25
Tertiary	102	25.50
Number of years of having hypertension		
≤10 years	318	79.50
11–20 years	49	12.25
21–30 years	20	5.00
31–40 years	12	3.00
41–50 years	1	0.25

### Experiences of side effects

The side effects experienced by patients using the HMSSES are presented in Table 2. These experiences are categorized as no/low (39.75 %), moderate (53.0 %) and high (7.25 %). From the qualitative interviews, participants mentioned palpitations, frequent urination, recurrent bouts of hunger, erectile dysfunction, dizziness, headache, cough, physical exhaustion and weakness. A female participant reported,

**Table 2:** Experiences of medication side effects

Side effect	Never	Rarely	Sometimes	Very often	Always
	*n* (%)	*n* (%)	*n* (%)	*n* (%)	*n* (%)
Dizziness	179 (44.75)	61 (15.25)	124 (31.00)	31 (7.75)	5 (1.25)
Fatigue	79 (19.75)	29 (7.25)	128 (32.00)	112 (28.00)	52 (13.00)
Cough	197 (49.25)	80 (20.00)	93 (23.25)	24 (6.00)	6 (1.50)
Headache	89 (22.25)	50 (12.50)	186 (46.50)	50 (12.50)	25 (6.25)
Confusion	201 (50.25)	102 (25.50)	88 (22.00)	8 (2.00)	1 (0.25)
Depressed mood	189 (47.25)	108 (27.00)	85 (21.25)	16 (4.00)	2 (0.50)
Chest pain	220 (55.00)	56 (14.00)	89 (22.25)	27 (6.75)	8 (2.00)
Difficulty breathing	251 (62.75)	49 (12.25)	75 (18.75)	17 (4.25)	8 (2.00)
Fainting	327 (81.75)	35 (8.75)	24 (6.00)	10 (2.50)	4 (1.00)
Constipation	142 (35.50)	97 (24.25)	117 (29.25)	28 (7.00)	16 (4.00)
Diarrhoea	206 (51.50)	105 (26.25)	77 (19.25)	8 (2.00)	4 (1.00)
Flu-like symptoms	156 (39.00)	130 (32.50)	80 (20.00)	25 (6.25)	9 (2.25)
Swelling in the ankle/feet	206 (51.50)	51 (12.75)	77 (19.25)	47 (11.25)	19 (4.75)
Increased frequency of urination	82 (20.50)	34 (8.50)	99 (24.75)	104 (26.00)	81 (20.25)
Reduced sex drive	86 (21.50)	47 (11.75)	105 (26.25)	56 (14.00)	106 (26.50)
Erectile dysfunction (impotence)	335 (83.75)	19 (4.75)	19 (4.75)	14 (3.50)	13 (3.25)
Rash	278 (69.50)	75 (18.75)	37 (9.25)	9 (2.25)	1 (0.25)
Difficulty sleeping	167 (41.75)	50 (12.50)	111 (27.75)	36 (9.00)	36 (9.00)

No = never, low = rarely, moderate = sometimes, high = very often and always. No/low side effects = 39.75 %, moderate side effects = 53.0 %, high side effects = 7.25 %

When I take the drugs I was given, in about 20–30 minutes my heart will be beating fast and even my whole body will be shaking …I urinate a lot and feel very hungry in the mornings. (R1: female).

Moreover, the concern of a male participant was expressed in the following:

I lose erection. That is the main thing that is worrying me so much. Sometimes I don’t perform well sexually, my wife complains and there’s a problem at home. (R5: male).

The participants perceived that their forgetfulness in taking the medicines, the lack of thorough laboratory investigations, the chemical composition of their medicines and the daily intake of the medicines were the main factors accounting for the side effects they experienced. These views have been reported by the ensuing comments:

We don’t have funds otherwise before a medication is administered to a patient a lot of laboratory tests have to be done so that we don’t have these problems. (R3: female).

The drugs are not natural. They contain serious chemicals and that is why we are suffering from the effect of the chemicals. (R5: male).

### Psychological symptoms and experience of side effects

The presence of symptoms of common psychological conditions like depression, anxiety and stress (Table 3) may influence the side effects experienced by patients (*n* = 400). Significant relationships between depression (*x*^2^ = 24.21, *p* < 0.0001), anxiety (*x*^2^ = 42.33, *p* < 0.0001), stress (*x*^2^ = 39.73, *p* < 0.0001) and side effects were observed. A logistic regression model using the adjusted results for the association between depression, anxiety, stress and side effects are shown in Table 4. Participants with depression, anxiety and stress respectively had a 1.9, 1.5 and 1.3 times increased likelihood to experience side effects than those without these psychological symptoms.

**Table 3:** Description of depression, anxiety and stress symptoms

Psychological condition	Normal	Mild	Moderate	Severe	Extremely severe
	*n* (%)	*n* (%)	*n* (%)	*n* (%)	*n* (%)
Depression	358 (89.50)	25 (6.25)	12 (3.00)	4 (1.00)	1 (0.25)
Anxiety	129 (32.25)	46 (11.50)	140 (35.00)	44 (11.50)	41 (10.25)
Stress	259 (64.75)	59 (14.75)	52 (13.00)	26 (6.50)	4 (1.00)

Group 1 (no/low symptoms) = normal + mild; group 2 (presence of symptoms) = moderate + severe + extremely severe

**Table 4:** Logistic regression model for depression, anxiety and stress symptoms in relation to the experience of side effects

Variable	Odds ratio	95 % CI	*p* value
Presence of depression (ref: no/low depression)	1.9	1.03–3.57	0.04
Presence of anxiety (ref: no/low anxiety)	1.5	1.22–1.77	<0.001
Presence of stress (ref: no/low stress)	1.3	1.02–1.71	0.04

### Medication side effects and adherence

Medication adherence behaviour of patients was assessed using MMAS (Table 5). After controlling for demographic characteristics (age, sex, education) and clinical characteristics (hypertensive years, comorbidities, number of medicines), the results indicated that side effects significantly increased the probability of non-adherence [OR = 4.84 (95 % CI 1.07–1.85), *p* = 0.04] (Table 6). Given this quantitative finding, the exploration from the qualitative interviews revealed that social factors, media influences and attitudes of primary caregivers further explained this relationship between side effects and adherence.

**Table 5:** Frequency distribution for Morisky Medication Adherence Scale

Question	Yes	No
1. Do you sometimes forget to take your hypertension medications?	Freq. *n* (%)	Freq. *n* (%)
	282 (70.5)	118 (29.5)
2. Over the past 2 weeks, were there any days when you did not take your hypertension medicine?	328 (82.0)	72 (18.0)
3. Have you ever cut back or stopped taking your medication without telling your doctor because you felt worse when you took it?	341 (85.25)	59 (14.75)
4. When you travel or leave home, do you sometimes forget to bring along your medications?	327 (81.75)	73 (18.25)
5. Did you take your high blood pressure medicine yesterday?	335 (83.75)	65 (16.25)
6. When you feel like your blood pressure is under control, do you sometimes stop taking your medicine?	340 (85.0)	60 (15)
7. Do you ever feel hassled about sticking to your blood pressure treatment plan?	337 (84.25)	63 (15.75)
8. How often do you have difficulty remembering to take all your medications?	Freq.	Percentage
○ Never/Rarely	6	1.50
○ Once in a while	6	1.50
○ Sometimes	34	8.50
○ Usually	101	25.25
○ All the time	253	63.25
Level of adherence		
Low	323	80.75
Moderate	50	12.50
High	27	6.75
Re-categorization of adherence level		
Poor	373	93.25
High	27	6.75

Low and moderate adherence = poor adherence

**Table 6:** Logistic regression model for side effects and non-adherence

Variable	Odds ratio	95 % confidence interval	*p* value
Moderate to high side effects (ref: no/low side effects)	4.84	1.07–1.85	0.04

Demographic and clinical characteristics were controlled

#### Social factors

Some participants experienced erectile dysfunction as a result of the medication side effects, and the desire to sexually satisfy partners came up as a hindrance to medication adherence. The concern of a male respondent was expressed as follows:

Sometimes I stop taking the medication because my wife is complaining I don’t perform well. If the drug problem is posing problems for me in the room what do I have to do; either I stop taking it or I fall on the supplementary ones. (R5: male).

#### Media influences

Some patients did not adhere as a result of information about medicines obtained from the media which caused trepidation. The fears of a participant were articulated as follows:

I don’t take my medicine all the time because I listened to a man on …. FM who said that some of the hypertension drugs can cause liver or kidney cancer. (R1: female).

#### Attitudes of doctors

The perceived negative attitude of primary care givers towards patients hindered the intention of some participants to adhere to medications as a result of the side effects. A male respondent did not adhere adequately because:

Our doctors have certain attitudes that are very obnoxious sometimes….. because if I go to the doctor and I say I think I have a problem with my medicine …, he will say who told me about it, who told me I have a fever. It’s like I am a novice. I will expect the doctor who knows more to allow me to pour all the nonsense I have concerning my condition so that he will pick out what is good from the nonsense that I have said. (R8: male).

### Coping with side effects

In coping with the problems associated with patients’ medication intake, three interrelated subthemes emerged from the interviews. Some patients personally adopted medication modification strategies; others espoused the use of complementary and alternative treatments, while some relied on the interventions made by their doctors.

#### Direct personal effort

Among patients who took direct responsibility to manage their problems, reducing the dosage of their medications and changing the times of taking their medicines were the main strategies they applied. By taking a lower dose of the medicine to minimize the negative effects, a female participant said,

When the doctor told me he will not change my medicine and I should still take the medicine like that, I decided to divide the tablet into two and I only take half of it. (R1: female).

Contrary to the information that diuretics were convenient when taken during the day, a patient thought it better to do otherwise. According to her,

I take my medicine at night so I can go about my day’s activities without having to urinate frequently. (R2: female).

#### CAM use

Complementary therapies were sometimes used by participants to manage the negative effects of their conventional medications:

I take a mixture of lemon and water three hours before taking my BP medication and I also use dandelion and moringa for food to help solve the problems with my medicine. (R1: female).

Additionally, alternative treatments were sought to replace medications with unwanted effects. This view was explicitly provided:

I use the natural way. I take fruits and feed myself on fruits alone for say one week without taking my medicine. When I do that I don’t feel anything bad about the medication. So I believe that the natural ways we were taught from the Bible saying we should take fruits helps because when I try it, it works for me. I take banana, oranges, and pineapples in the morning, maybe vegetables in the afternoon and fruits in the evening. (R5: male).

#### Intervention by doctors

Some participants further indicated the timely intervention by their doctors as serving to deal with medication challenges. Patients usually had the medications with the side effects changed by their doctors. According to one participant:

I brought the medicine to the doctor at the next visit and he changed it for me. (R3: female).

Similarly, a female participant admitted,

Formerly I was put on nifedipine and it came to a time when I take it I feel severe headache so I complained and the doctor changed it. (R10: female).

Likewise, another patient said,

I was given lisinopril and when I take it I cough a lot so I told the doctor and he changed it for me. (R13: female).

## Discussions

The majority (60 %) of hypertensive patients pointed out that they experienced moderate to high side effects which included difficulty sleeping, erectile dysfunction, reduced sexual drive, constipation, chest pain, depressed mood, headaches, cough, fatigue and dizziness. These symptoms are consistent with what has been previously reported and clearly outlined in medical literature [6] and provide evidence that side effects play an important role in the management of hypertension because of its association with adherence to recommended remedies. The present study observed that medication side effects potentially repressed adherence, and this role of side effects as a major cause of non-adherence in both new and old patients with hypertension is corroborated [8, 14–17].

In comparison with earlier investigations on medication side effects, the current study has also shown that the roles of cognition, society, the media and the attitude of clinicians were important in medication adherence behaviour [18, 19]. First, remembering to take one’s medication is a vital tool in aiding adherence to conventional treatment [20]. Second, there is the heightened need for clinicians to consider educating sexual partners of male respondents who stop taking their medications because they want to avoid the problem of erectile dysfunction. Similarly, experiences of medication side effects had psychosocial underpinnings. For example, though the feeling of erectile dysfunction is a biological issue, the thought of becoming impotent has a strong psychological impact on a man within the African cultural context [21]. Socially, the need to be able to satisfy sexual partners would be an important issue of concern. This confirms that social and behavioural issues are critical to clinical care [22] and the need for emotional support to improve adherence behaviour [23].

Media censoring can help to curb the influence of superficial health information on the adherence behaviour of patients. Listening to information on medication and their side effects led to non-adherence among some participants. Yet the media could be probably explored as a helpful tool to send important information highlighting medication adherence [24].

The presence of common psychological problems such as anxiety, depression and stress contributed to the extent to which the participants experienced the side effects of their medications. Hypertensive patients experience intense emotions which can increase their risk for the development of mental health disorders particularly anxiety and depression, and these symptoms increase their susceptibility to the negative effects of the medications [25].

To reduce the unpleasant nature of the side effects, some participants were motivated to modify the dose or the timing of medication intake, or have their regimen amended by their clinicians. On the other hand, some patients sought to use complementary and alternative therapies. If, in contrast, the chemical composition of the medications were perceived to be causing the adverse effects, it could be argued that the use of CAM was probably based on the perception that CAM remedies are natural, less toxic and have relatively little or no side effects [26].

This study acknowledges the limitation related to the representativeness of the hypertensive patient samples from the study sites which were both tertiary health institutions. Although we collected data from the two major institutions located in different regions of the country, hypertensive patients are managed at other levels of healthcare and information on such patients were not obtained in the study.

## Conclusions

Results from this study show that, in addition to a biomedical approach, the experience of side effects has biological, social and psychological interrelations. The result offer more support for the need for a multi-disciplinary approach to healthcare where all forms of expertise are incorporated into health provision and patient care. Thus, addressing this problem from a biopsychosocial perspective in any intervention may improve adherence and invariably control blood pressure.
